# Context-Aware Personal Navigation Using Embedded Sensor Fusion in Smartphones

**DOI:** 10.3390/s140405742

**Published:** 2014-03-25

**Authors:** Sara Saeedi, Adel Moussa, Naser El-Sheimy

**Affiliations:** Department of Geomatics Engineering, University of Calgary, 2500, University Drive, NW, Calgary, Alberta T2N 1N4, Canada; E-Mails: amelsaye@ucalgary.ca (A.M.); elsheimy@ucalgary.ca (N.E.-S.)

**Keywords:** context-aware, personal navigation service, sensor fusion, activity recognition, vision-aided navigation

## Abstract

Context-awareness is an interesting topic in mobile navigation scenarios where the context of the application is highly dynamic. Using context-aware computing, navigation services consider the situation of user, not only in the design process, but in real time while the device is in use. The basic idea is that mobile navigation services can provide different services based on different contexts—where contexts are related to the user's activity and the device placement. Context-aware systems are concerned with the following challenges which are addressed in this paper: context acquisition, context understanding, and context-aware application adaptation. The proposed approach in this paper is using low-cost sensors in a multi-level fusion scheme to improve the accuracy and robustness of context-aware navigation system. The experimental results demonstrate the capabilities of the context-aware Personal Navigation Systems (PNS) for outdoor personal navigation using a smartphone.

## 1. Introduction

The emergence of wireless communication and mobile devices equipped with global positioning system (GPS) ignited the idea of personal navigation systems (PNS). PNS includes positioning capability and navigation functions to provide location information using portable devices for individuals. Context-awareness is an emerging research topic in the area of PNS. Context-aware systems take into account contextual information in order to adapt their operations to the current context without explicit user intervention. The phrase “user context” is characterized by the situation of the user in terms of his/her activity, location, preferences and environment [1]. Useful context information in PNS is related to the user's activity (e.g., walking, driving) and the device placement. Such contextual information can provide context-specific services for PNSs. In PNS, the user's mobility necessitates an adaptive behavior according to changing circumstances such as in-vehicle or on walk modes [2]. Moreover, unlike other navigation systems, a mobile device is not held in a fixed position and can spontaneously move with the user. When processing multi-sensor data in a PNS, sensors’ placement impacts the positioning solutions. Since the mobile device is either mounted on the body or carried by the user in hand, the orientation output of a mobile device depends on its placement with respect to the user. One approach to overcome this issue is to identify the user activity and device placements and customize the navigation solution using the recognized context information.

With the advances in micro-electro-mechanical system (MEMS) sensor technologies on mobile devices (e.g., accelerometer, gyroscope, magnetometer), collecting a vast amount of information about the user is feasible in an automatic way; however, it is still difficult to organize such information into a coherent and expressive representation of the user's physical activity [3,4]. In other words, there is a gap between low-level sensor readings and their high-level context descriptions. The main objective of this paper is developing a context-aware system which robustly recognizes user activity and device placement based on fusion of smartphone's low-cost sensors and then, adapting the pedestrian navigation solution based on the user's contexts.

There are a few studies aimed at supporting PNS computations using context information [5–7]. This research is one of the original works in supporting the personal navigation services by providing context information. This paper contributes to the intelligent PNS area in the following three aspects:

- Sensor integration: As the accelerometers are usually embedded on the mobile devices, most of the existing activity recognition systems use only accelerometers and rarely consider fusion of other sensors [5]. As an improvement to the previous works, accelerometer, gyroscope as well as magnetometer sensors are integrated to recognize activity context more reliably. Moreover, in most of the research works in this area, the device is fixed to the users’ body or has a predetermined orientation. However, in this paper no assumption is made about how users carry their mobile phones.
- Context detection algorithm: The most advantageous methodology for context detection is fusing multi-sensor and multi-source data. Since the context information may be acquired from heterogeneous sources, defining an appropriate strategy to integrate various sources of information is necessary. Therefore, a hybrid multi-level context detection algorithm is developed to integrate data-driven fusion at the signal level and knowledge-driven fusion at the decision level. Moreover, fuzzy inference engine is used for uncertainty modeling of the hybrid method. This algorithm provides more reliable and readable method which is less sensitive to the noise of the signals.
- PNS application: One of the main contributions of this paper is development of a context recognition algorithm for vision aided GPS navigation of a walking person while holding the smartphone in different orientation. This is an original work in PNS which improve the vision aided navigation solution using context information. By using context information, the vision-based algorithm can be aware of the appropriate user mode and the device orientation to adapt detection of the velocity and orientation changes using visual sensor.

## 2. Background and Related Works

Context-aware applications use context information such as user's activity to evaluate the user and/or the environment situation and then reason about the system's decisions based on the context information. While different methodologies have been studied for the automatic recognition of human activities context and environmental situation for various context-aware applications (e.g., health-care, sport, and social networking [8–11]), this study is one of the first works that applies user activity context in PNS and specifically in vision-aided navigation. A new hybrid paradigm is introduced for context recognition and applying the context for PNS application. In navigation applications, the useful context includes the user's activity (e.g., walking, driving) and the device placement and orientation.

The research literature in activity recognition using multi-sensor information focuses on two types of approaches: data-driven and knowledge-driven paradigms. Data-driven paradigms which employ the fusion of different sensors typically follow a hierarchical approach [11]. First the sensors’ providers collect and track useful data about the user's motions. The next step is to extract features and characteristics of the raw measurements using statistical techniques. Finally, a machine learning algorithm is used to recognize the user's activity based on the comparison of the extracted features with those that are already extracted for each mode [5]. These techniques are used for simple and low-level activities and differ on the number of used sensors, considered activities, adopted learning algorithms, and many other parameters. The accuracy of the data-driven techniques depends mainly on the complexity of the activities, availability of the sensor data, finding the optimum features, accuracy of the training sets and using the best machine learning method for the specific application. Therefore, to detect the low-level activity contexts, different sensor signals, features sets and classification techniques are examined in this research to find the optimum data-driven context detection algorithm for PNS. The accuracy of this data-driven method is further improved by using high-level knowledge-driven algorithms. As an improvement to the previous works, accelerometers, gyroscopes and magnetometers are integrated to recognize activity context more reliably. Moreover, in most of the research works in this area, the specialized accelerometers are fixed to the users’ body or have a certain orientation. This assumption usually does not hold for the usual case of carrying the phone in the hand or pocket. However, in this study no assumption is made about how users carry their mobile phones.

Knowledge-driven paradigms for reasoning about human activity have been investigated in ubiquitous computing and artificial intelligence. Various formalisms are developed in this respect and these methods differ in the expressiveness of the logic, the implicit or explicit representation of contexts, and the complexity of reasoning [12]. Recently, logic-based knowledge representation formalisms have emerged in activity reasoning because of their high expressiveness combined with desirable computational properties [12]. Also, ontological approaches have been used for activity modeling in order to define the formal semantics of human activities by means of the operators of the ontological language. Then, ontological reasoning is used to recognize that a user is performing a certain activity using some evidences (e.g., sensor data, location of persons and objects, properties of actors involved). Most of the knowledge-driven techniques have been used in pervasive computation for reasoning about the environmental situation and inferring complex and high-level activities such as sleeping, brushing teeth, cooking or being in a meeting. To improve the data-driven classification, a rule-based engine is developed to reason about higher-level activities by incorporating GPS and temperature sensors as well as correlation of walking pattern. A high-level decision-level fusion is applied to detect high-level context information from multiple information sources. This paper is one of the first works in navigation context detection which use a fuzzy based decision-level fusion algorithm in combination with a statistical data-driven recognition technique to improve the accuracy and consistency of activity context.

To integrate the advantages of both data- and knowledge-driven approaches, hybrid mythology is recommended in pervasive computation research field [13]. A hybrid knowledge-driven approach is proposed in [8] for real-time and continuous activity recognition using multi-sensor data in smart homes. This study makes extensive use of domain knowledge in the life cycle of activity recognition based on an ontology-based semantic reasoning and classification. In another work [14], knowledge based Bayesian Network was examined to incorporate prior knowledge to reduce the amount of training data. A constrained structure learning method is used in this study to learn activities such as walking, running, leaving and entering car, and *etc.* using camera stream. In another work [15], ambiguity learning has been developed to detect basic activities such as walking and running using accelerometer and GPS sensors on a smartphone for health-care purposes. This method is based on a logic-based framework to decompose complex activities into simpler ones and integrate it with machine learning techniques to process sensor data. However, one limitation in almost all of these techniques is that there is no support for imperfect information and consideration of uncertainty. Uncertainty is an integral part of the activity recognition and is mostly caused by the imperfectness and incompleteness of sensed data. Hence, to have more reliable results, the modeling of uncertainty is an essential step. This research improves the previous hybrid methodologies by using fuzzy inference for uncertainty modeling. The proposed methodology aims at integrating the data-driven paradigm with the knowledge-oriented paradigm to solve activity detection problems. Fuzzy inference system is used to transform the data into higher-level descriptions of human activities for uncertainty modeling and considering experts’ rules and other information sources. This method is capable of handling the uncertainty of the signal processing activity detection, removing the conflicts and preserving consistency of detected activities, filling the gaps, and fusing various sources of information.

Conventionally, acceleration sensors have been applied in most of the physical activity recognition research works because they are small, inexpensive, light-weight, and consume little power [16]. However, the fusion of multiple sensors including several accelerometers, gyroscopes, GPS, camera, and infra-red (IR) motion detectors not only improves the results but is rather mandatory for an accurate activity recognition system, as noted by Kern, *et al.* [1]. In this research, a smartphone such as Samsung Note1 is employed as a multiple sensor device to recognize the interesting activities for PNSs. Smartphones have been recently used in monitoring human activities because of their portability (small size and light weight), considerable computing power, embedding of various sensors, ability to send and receive data, and their increasing popularity. A wide variety of research works has been conducted in activity recognition using smartphones [3,5,9,17]. As the smartphones are limited in terms of energy and computing power, different approaches have been suggested to overcome such a challenge. In the first group of studies, the complete recognition procedure can be done on the mobile device [10]. This needs simplified recognition process and hardware-friendly approaches designed for pattern recognition techniques [9]. In the second group [3,5,17], a portion of computation is done on the mobile phone and the other part can be done on a central location or a server computer. These systems send the data to the server and the recognition process is performed on the server, then the results are sent back to the mobile phone. In this case, calculation capacity would not be an issue, but on the other hand, data transfer is the problem. In this research, we used the client-server architecture to employ more computing power in order to find the best set of sensors, features and learning algorithm and then, designing the most accurate and fastest approach for detection of navigation contexts. However, computations of the activity context-recognition can be performed on the mobile client. Moreover, this is one of the first research works in the field of activity recognition using smartphone which attempts to consider a variety of sensors (e.g., accelerometer, gyroscope, magnetometer, GPS and temperature) and to find the best set of sensors for a specific application.

## 3. Context-Aware PNS using Multi-Level Sensor Fusion

**Figure 1.**
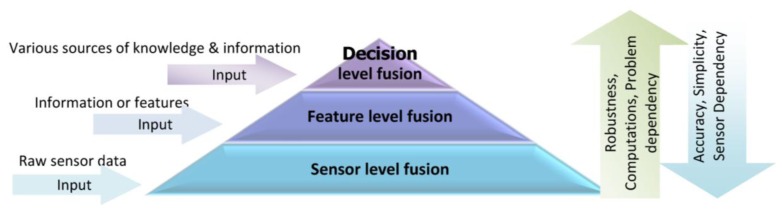
Multi-level sensor fusion pyramid.

Information gathered by a single source is usually limited and may not be fully reliable, accurate and complete; therefore, the proposed approach in this research is using multi-sensor data in a multi-level fusion scheme to improve the accuracy and robustness of context-aware PNS. Multi-sensor fusion is the process of synergistic combination of evidences from different sources to provide a better judgment. One of the important issues concerning information fusion is to determine how this information can be integrated to produce more accurate outcomes. Depending on the stage at which fusion takes place, it is often divided into three categories: sensor level, feature level and decision level [18]. Figure 1 shows a multi-level sensor fusion pyramid along with the input of each level.

The choice of a suitable fusion level depends on information type and applications. In sensor or low-level fusion, the integration techniques works directly on the raw measurements obtained from sensors. Feature or median-level fusion works on the extracted features which are available from different sources of information. Feature-level fusion is an appropriate level when features are provided from different sensors. Decision level fusion techniques take place at the decisions and interpretations from different knowledge sources. These techniques are more suitable when information sources are naturally diverse. The comparison of fusion techniques in different levels is listed in Table 1. As there is no simple rule for selecting the proper fusion technique, a wide range of integration techniques are potentially applicable for each fusion level.

**Figure 2.**
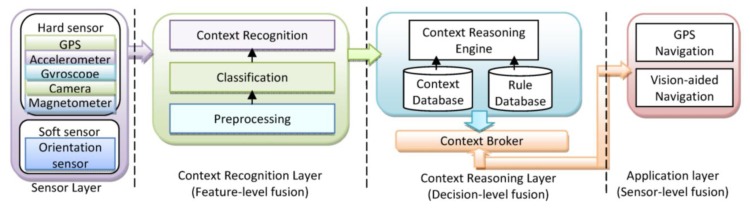
Schematic diagram of the context-aware navigation services architecture.

A context-aware system is concerned with the context detection, context reasoning and application adaptation based on the recognized context. In this paper, different techniques and models will be used for fusion in different levels. Sensor level fusion is used in location determination; feature level fusion is illustrated in activity recognition; and decision level fusion is applied for context reasoning to infer the context information. The application of the fusion approaches show success with techniques ranging from probabilistic theory to fuzzy and expert systems. In Figure 2, simplistic system architecture of a context-aware PNS is shown.

## 4. Context Recognition using Feature-Level Fusion

**Figure 3.**
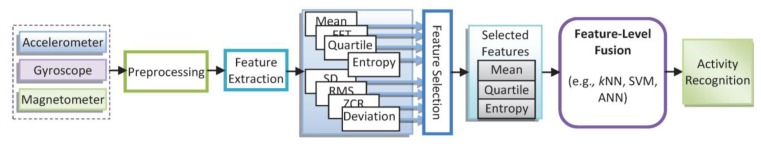
Activity recognition procedure using feature level fusion.

The primary contexts relevant to the mobile navigation services can be divided into two categories: user activity, and device placement. User activity context (e.g., stationary, walking, running, ascending/descending stairs, using an elevator) refers to a sequence of motion patterns executed by a single person and at least lasting for a short duration of time. Another important context in PNS is that “where the device is located with respect to the user”. Usually a mobile device can be carried out by the user in an arbitrary placement (e.g., on the belt, in the pocket, in the bag, in the hand for talking, texting or while arm swing). Although there is a wide variety of research in activity recognition using wearable sensors; a limited studies use a mobile device to collect data for activity recognition [3,5,6,17,19]. Although the activity recognition results in almost all of these research works are promising; however, they didn’t consider the impact of carrying the phone in different placements and diverse range of activities. Inspired by these results, we have used accelerometer, gyroscope and magnetometer sensors to consider both motion and orientation of the device. To recognize context information, a feature-level fusion scheme is applied using the extracted features from each sensor instead of the raw sensor data. Since the feature set contains richer information, integration at this level provides better recognition results. As the fusion does not use the raw sensor data, the scalability and sensor independency is increased; however, it requires transforming data to an appropriate feature space. Figure 3 demonstrates the procedure of feature level fusion which integrates multi-sensors data.

As shown in Figure 3, the raw data captured by sensors is pre-processed for calibration and noise reduction. Then, signal processing algorithm is used to derive an appropriate set of features from the measurements. The potential number of features that can be used is numerous; however, the used features need to be carefully selected to perform real-time and robust context recognition. After feature extraction, classification techniques can be used to classify the feature space. There is a wide variety of feature extraction and classification techniques; and often selecting the best one depends on the application [20]. In this research the following features (Table 2) has been used in time and frequency domains for context detection based on inertial data.

These features are used as inputs in the classification methods, namely Bayesian Network (BN), Naïve Bays (NB), and Artificial Neural Network (ANN) which are listed in Table 3. In order to select the best technique, these classifiers have been evaluated using various datasets by applying Waikato Environment for Knowledge Analysis (WEKA), a free popular software for machine learning algorithms written in Java, developed at the University of Waikato, New Zealand toolbox [21]. The details of classification methods can be found in [22].

### 4.1. Experiment and Results

To find the optimum set of sensors and features that contributes to an accurate context detection algorithm, an activity recognition module is developed. Using the activity recognition module and extensive experiments, the performance of activity recognition module has been evaluated for different user's modes and motions. A Samsung Galaxy Note 1 smartphone was used for the purpose of data collection for this study. The software architecture of the proposed context-aware model for navigation services is client-server architecture. In this architecture, application logic can be split between the local android device and a server-side resource that can tap into larger databases and computing power. For example, recorded accelerometer data on the local android device are preprocessed and the mean value of each sample window sent to a web server where the data is compared against a database of context patterns. Using the Wi-Fi networks, the data can be synchronized with the server immediately. To gather data from the phone, an application is developed to capture and send the data to a server computer. This application can be used in real time and collects data with a timestamp. End users access applications through a light-weight mobile application while the main software and user's data are stored on servers at a remote location. All required sensor data for detection is pre-processed and sent to the server automatically or by the user push. In the next step, after processing data for context detection and finding the appropriate navigation solution, the results are sent back to the mobile user. Activity data was collected from four subjects consisting of two males and two females, their age ranging from 26 to 40. In order to collect the test data, the smartphone was loosely placed in different orientations including in the bag, in the jacket pocket, on the belt, in hand close to ear for talking, and down at one's side while arm is swinging. No special requirement has been imposed on how to wear the smartphone except for its location on the body. Each activity with a different device placement mode was performed for two minutes and stored in the database (DB) on the server. To build the reference data, subjects were asked to annotate main activities with start and finishing times.

**Figure 4.**
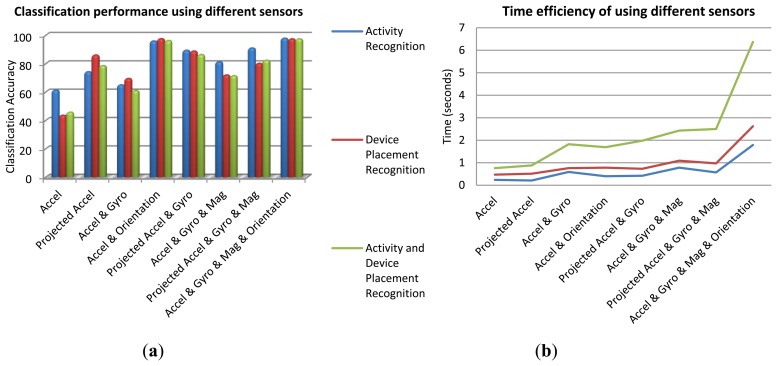
Results of contexts recognition using BN classifier and all the seven features: (**a**) Overall accuracy of different sensors; (**b**) Time consumption of different sensors.

The best set of sensors for activity recognition is the ones with the highest correlation with the activity classes. Accelerometer sensors have been widely used for motion detection. Gyroscope is useful for capturing user's motion and device orientation changes. Orientation determination is a significant feature to distinguish among sets of on-body device placements and determining the device orientation in each placement. Magnetometer sensor also helps determining the orientation as well as absolute heading information. In addition to such physical hardware-sensors, orientation software-sensor (or soft-sensor) provided by android API can be used to estimate the device orientation. This sensor fuses three accelerometer, gyroscope and magnetometer sensor signals to output the orientation angles (roll, pitch, and yaw). These angles describe the orientation of the device coordinate system with respect to the local navigation reference frame. The output of the orientation soft-sensor can be either used as an independent sensor or as a means to project other sensor data from device's coordinate system to the reference navigation system. The results of context recognition using different sensors have been investigated for the whole dataset. Figure 4a gives the overall classification accuracies of the three recognition scenarios: user's activity, device placement, and both activity and device placement. In this investigation, a BN classifier was applied using all the features. Time efficiency is a critical issue when using smartphones. Figure 4b shows time efficiency obtained from different sets of sensors for the DB of all users and all activities. Although this figure is showing the time consumption for a specific computer, it is useful for comparing the time efficiency achieved by using different sensors. By comparing Figure 4a,b, it is obvious that although applying all the sensor information leads to the highest accuracy, using accelerometer and orientation information has a better balance between accuracy and battery consumption.

After selecting the appropriate sensors, the data is divided into two-second segments and features are extracted from 80 readings conducted within segments. The two-second duration is chosen because the experiments show that it provides sufficient time to capture meaningful features involved in different activities. The signal windows have 50% overlap. To investigate the feature extraction, various combinations of sensors are considered for discerning each set of activity and device placement. To increase robustness of activity recognition and reduce computations, a SVM (support vector machine) and gain-ratio based feature selection method is applied and a set of four features has been selected with the same level of accuracy for classification approach [23]. Table 4 lists the best set of features selected using SVM and gain-ratio feature evaluator. Also, the corresponding recognition accuracy using BN classifier for each set of features is mentioned in that table.

Comparative studies on classification algorithms are difficult due to the lack of universally accepted quantitative performance evaluation measures. Many researchers use the classification error (overall accuracy) for quality measurement; therefore, this research adopts a similar approach [23]. The 10-fold cross-validation is used to evaluate the classification models. After each folder is tested, we compute the average classification error of all the folders as the overall accuracy.

**Figure 5.**
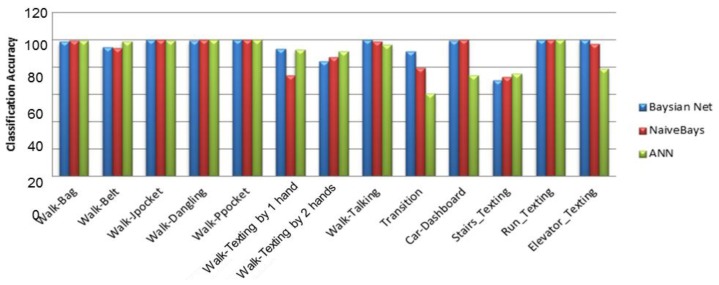
Recognition accuracy rate using different classifier for different activity modes-feature space: four essential features selected by SVM feature evaluator method.

Figure 5 shows the context recognition rate for BN, NB and ANN classifiers using four features selected by SVM feature evaluator. By investigating each activity's recognition rate, it can be inferred that the classification models distinguish between the device placements and user activities with an overall accuracy of 95%. Although ANN requires more computational capabilities in comparison to BN and NB methods, the accuracies obtained from the three classifiers are close to each other (Table 5). This could be the result of the fact that the activities are discriminated by the four extracted features with a high accuracy.

To improve efficiency of the feature-level fusion and establish a good balance between accuracy and computational cost, the optimum set of sensors, features and the best classifier have been selected. Results showed that using accelerometers are efficient in recognition of user motions, but not enough for recognition of device placement; therefore, we added the orientation soft-sensor (based on the fusion of accelerometer, magnetometer and gyroscope), which can relieve the effect of the orientation changes on the performance of activity classification. Experiments conducted for feature selection demonstrated that when feature selection methods were applied, it was successful in removing redundancy in features and thus reducing computations. For activity recognition, four features have been chosen instead of all the features to reduce computational load without compromising accuracy. Compared to the more complex classifiers such as ANN, the results showed that the BNs yielded a similar performance, having a more extensible algorithm structure and requiring fewer computations. The BN classifier provides an overall recognition accuracy of 84.95% on a variety of six activities and six device positions using only four features provided by SVM feature selection method.

## 5. Context Reasoning Using Decision-Level Fusion

**Figure 6.**
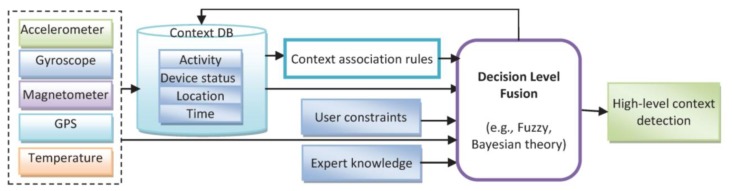
Context reasoning using decision level fusion.

Using the results of the previous section, it can be observed that some of the activities (such as walking and using stairs) and some of the device placements (such as on-belt and trousers front pocket positions) were misclassified or cross-classified. This can be improved by reasoning about context information in higher level decision fusion using new information sources such as walking patterns and user's constraint. Context reasoning is required to handle the uncertainty of the recognized activities, remove the conflicts, preserve consistency of detected context, fill the gaps, and fuse various sources of information [24]. The correlation which exists in user's motion, environment and device orientation persuades mining the association rules between them. Then, combining these rules using a decision level fusion algorithm may generate a more powerful understanding of the current situation. “*Primary*” contexts, including location, activity and time, and fusion of them in the decision level might generate valuable knowledge which acts as a guide into other sources of contextual information [5]. For example, knowing the current location and current time, the system could have a pretty good idea of the user's current activity which can be used in context detection by adding association rules. In this research, after determination of location and recognition of activities, high level contexts are detected by incorporating association rules between the primary contexts in a reasoning engine. The decision level fusion applied in context reasoning engine improves efficiency of context detection algorithm by applying new rules which is acquired from various source of information such as historical context information, expert knowledge, user preferences or constraints. Figure 6 shows a decision level fusion which integrates heterogeneous source of knowledge, information and sensors. Context reasoning consists of context DB and context reasoning engines. The context reasoning engine infers deduced contexts, checking the consistency and monitoring the context information. Sensed and inferred context data can be converted to useful information according to the inference rules.

### 5.1. Fuzzy Inference System (FIS)

Fuzzy Inference System (FIS) is a method in which the parameters that influence the decision making process can be fused using a human like reasoning strategy. This is achieved by defining the so called linguistic variables; linguistic labels and membership functions [25]. The fuzzy reasoning process is then realized using the fuzzy if-then rules that enable the linguistic statements to be treated mathematically [25]. In this research, FIS is applied to model the context uncertainty and incorporate new source of knowledge using human rules. FIS was used for device placements and indoor/outdoor environment detection. The proposed linguistic variables (Table 6) can be obtained from different sources of information such as GPS, temperature and GIS (Geographic Information System) analysis.

**Figure 7.**
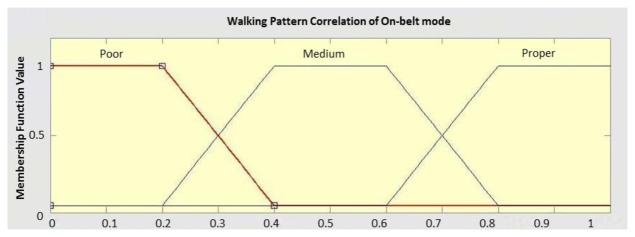
Fuzzy trapezoidal membership function defined for the walking pattern correlation.

For each of the linguistic variables, membership functions are defined by an experienced person. For example, Figure 7 shows the membership function defined for the walking pattern correlation which is a trapezoidal function. To have a fuzzy definition for the concept of context extraction, an output membership function must be defined as well. The output membership functions take different contexts as linguistic variables. It varies between 0 (stating that the context cannot be detected) and 1 (denoting perfect context detection possibility).

**Figure 8.**
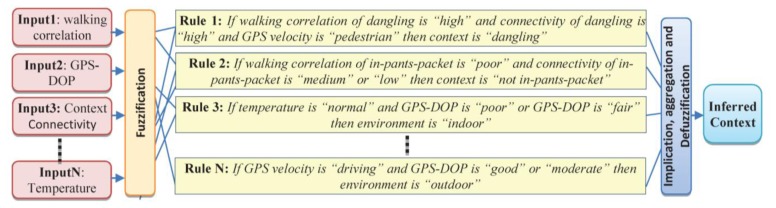
Evaluation of fuzzy rules using FIS.

Having determined the linguistic variables and corresponding membership functions, the next step is to determine the fuzzy rules between the input and the output membership functions. These rules are generated from association rules of the context DB and modified by an experienced person [20]. Based on the defined membership functions and the rules, fuzzy reasoning for the conjugate point determination is carried out in a Mamdani type fuzzy reasoning structure [25]. In the following four sample rules for detecting context information are presented in Figure 8.

Selecting the relevant input-output variables and an appropriate set of rules has direct influence on FIS performance. In designing rule repository, specific constraints can be defined to incorporate common-sense knowledge. This reduces the amount of required training data and makes the rule mining computationally efficient. An example of such a constraint is that a person cannot drive while in an indoor environment. Therefore, the rule repository is composed of a number of predicates generated by the user and designer along with the mined association rules. In the rule based engine, different types of rules have different levels of confidence and reliability.

### 5.2. Experiment and Results

**Figure 9.**
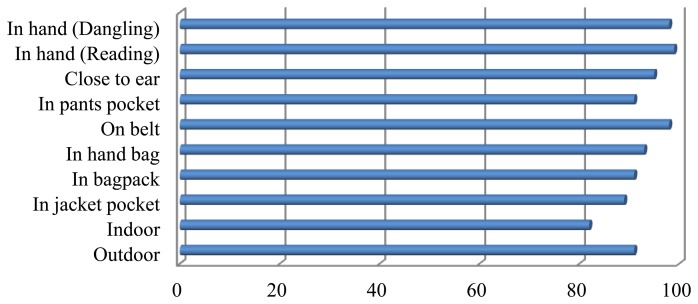
Recognition rates for different device positions using FIS algorithm.

After using FIS the results of context recognition is shown in Figure 9 for the same dataset. This figure depicts that using a context reasoning engine the overall accuracy for different context information has enhanced significantly. Feature-level fusion has the learning capability from sample datasets. Results of this paper show that feature-level algorithms are efficient in recognition of user motions, but inefficient in recognition of device location and orientation. One of the drawbacks of feature-level fusion is that they are not good for human interpretation of their internal representation and they cannot use human-like rules. On the other hand, most of these methods are based on the probabilistic assumptions of conditional independency and Gaussian distribution of probability distribution function. In contrast, decision-level fusion using fuzzy inference engine is based on human readable fuzzy rules. Context reasoning engine can improve consistency of decisions and handle uncertainty of classification using appropriate rules. This method is also efficient in computations. However, the construction of fuzzy rules & the determination of membership functions are subjective.

To cope with uncertainty in context detection, the hybrid method was investigated using a combination of learning based on BN and explicit rules written in possibility using FIS. The accuracy of the hybrid method is better than each method and is 97%, which is very promising (Table 7).

## 6. Location Determination Using Sensor-Level Fusion

**Figure 10.**
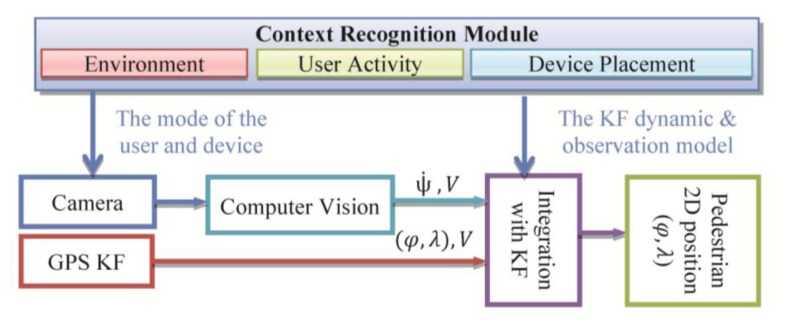
Multi-sensor pedestrian navigation diagram using context-aware vision-aided observation.

PNS requires continuous location determination and tracking of a mobile user with a certain accuracy and reliability. Detecting location of a pedestrian user is a very challenging task as (s)he moves in spaces where the usual positioning methods cannot work continuously in standalone mode. Location determination in indoor and outdoor environments is the main challenges towards building a ubiquitous PNS. Indoor/outdoor positioning technologies based on multi-sensor system including satellite and terrestrial positioning techniques is addressed in various research works [6,26,27]. Although positioning technologies is widely argued in the past few years, developing a system that enables ubiquitous location determination is still open for researchers. Most of the research work attempts to integrate multiple sensors/system [28]. Various sources of information can be loosely integrated to estimate position and orientation of the device in a way that is more accurate and pervasive than any of the individual sensors. In this research a context-aware system is developed to integrate measurements of different sensors using a sensor-level fusion. In this system, multi-sensor fusion is examined using camera and GPS sensors embedded on a smartphone. This system uses an integrated approach based on *Kalman filter* (KF). The details of the system can be found in [29,30]. Figure 10 shows the architecture of the location determination system which uses KF as the sensor-level fusion algorithm.

The design of the integrated pedestrian navigation algorithm is shown in Figure 10. When processing vision-based navigation measurements, sensors’ placement impacts the solution. Since the mobile device is either mounted on the body or carried by the user in hand, the orientation output of a mobile device depends on its placement with respect to the user. One approach to overcome this issue is to identify the user activity and device placements and customize the navigation solution using the recognized context information. The contexts that are useful for vision-aided system include: device orientation (face-up/down, vertical or portrait), device location (texting with one/two hand(s)), activity of the user (walking) and environment (indoor/outdoor). By texting we refer to the position of the user while texting and therefore it includes all similar positions such as surfing, playing, reading and *etc*. Texting mode requires the user to hold the device in front of her/him using one or both hands. The heading estimation from visual camera has been calibrated for this mode. Moreover, the heading measurements from visual sensor are compensated for the cases which the orientation of the device is changing instead of the orientation of the user by using the device orientation context. Also, the estimation of the velocity from camera is improved by a scale factor which changes adaptively based on the user mode such as walking, stairs, and running context information. The other context that can be used in this case is the environment (indoor/outdoor). This is used for selection of the sensors and definition of the device dynamic and observation model in the KF. For example, in the case of outdoor environment, GPS measurements can be used if its accuracy is acceptable.

When the context is changed, this triggered the context-aware PNS services to be refreshed by the update context information [22]. In this research as it has been mention in [22], an ontology-based model is developed using *web ontology language* (OWL). Context ontology applies axiom such as owl:subclass, owl:inverseOf, owl:unionOf, owl:disjointWith and owl:sameAs which are provided in OWL as shown in the following example:
<owl: Class rdf:ID=’Walking’> <rdfs:subClassof>  <owl:Restriction>   <owl:onProperty rdf:resource=‘Step_length’/>   <owl:toClass rdf:resource =‘#UserActivity’/>   <owl:classifiedAsrdfs:resource‘ftp://305678/classification#Alph_Reference’/>  </owl:Restriction> </rdfs:subClassof></owl:Class>

The ontology-based context metadata is generated using Protégé-OWL editor and is stored in the repository and retrievable by the inference engine.

### 6.1. Vision-Based Pedestrian Navigation Using Computer Vision Algorithm

Vision sensors are ideal for PNS since they are available in good resolution on almost all smartphones [29,30]. Vision-aided navigation has already been used for decades in navigation of robots; however, deploying it in pedestrian navigation has become a research topic only in the last few years [31,32]. The vision sensor is used to capture the user's motion using consecutive image frames in real time (also known as visual odometry). Device's displacement and orientation is estimated based on a real-time motion estimation of a single camera moving freely through an environment [32]. This system doesn’t need any special infrastructure and makes use of camera as an ideal aiding system.

This estimation can be useful in position and heading estimation of pedestrians; however, there are various issues when processing the video frames from a hand-held device's camera. Firstly, the measurements are relative; thus, estimation of absolute quantities needs initialization of the navigation parameters such as initial value for position and orientation. Furthermore, the observation scale cannot be obtained using only vision system and another sensor or a known dimension reference has to be used in order to retrieve the scale of the observation. Another issue is that the orientation of the mobile device affects the heading and velocity estimation of the camera. To overcome these issues, a context-aware vision-aided service is proposed to provide navigation solution using fusion of GPS and vision sensor. This service is aware of user and device context to use appropriate algorithm for each case to fuse navigation sensor data at a sensor level. For example, when the context information shows that device is in “texting” or “talking” mode, the observation from camera can be used for navigation. A KF is used to improve the navigation solution when the user is walking and the phone is in his/her hand. This method is based on the integration of the user's relative motion estimation (changes of velocity and heading angle) based on the video frame matching and absolute position information provided by GPS.

**Figure 11.**
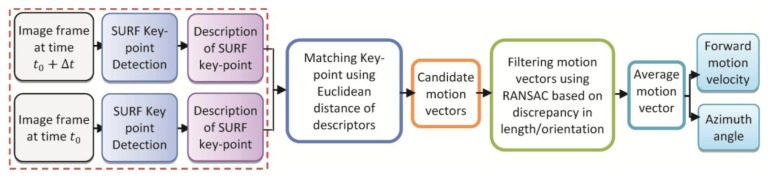
Flow-chart of the computer vision algorithm.

In this paper, a computer vision algorithm is developed to find the motion vector between two successive frames using an image feature matching algorithm. The motion vectors between two successive frames are then employed for estimation of forward motion velocity and the azimuth rotation angle. Motion estimation from video is a well-studied research in computer vision community [33]. Recently, a variety of local invariant descriptors have made remarkable progresses for motion estimation such as Speeded Up Robust Features (SURF). This method show excellent performance for image transformations, scale changes, rotation, blur, and illumination changes, *etc.* [34]. To detect the motion vectors, interest (key) points are detected from the frames using SURF algorithm. SURF key points are extracted in both successive frames, and then, the bidirectional nearest neighbor method, RANdom SAmpling Consensus (RANSAC) method [35] and dominant line direction method are used to realize the coarse-to-fine matching of key points [34]. The algorithm is shown in Figure 11.

**Figure 12.**
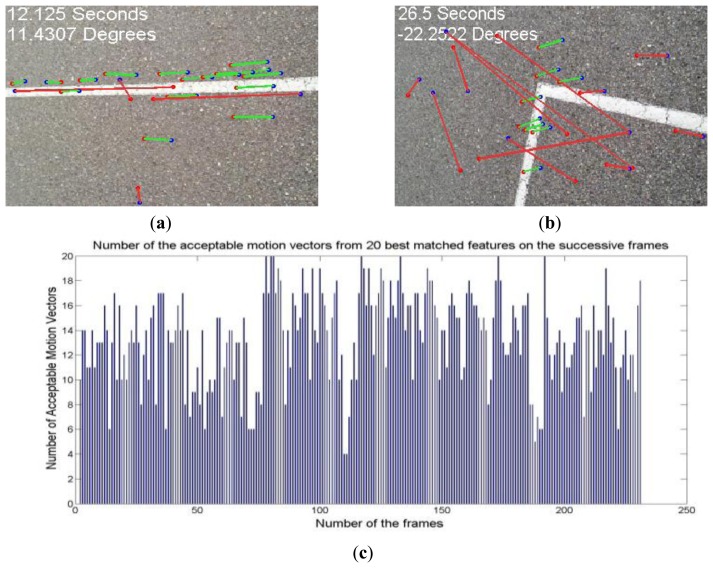
The matched features, candidate motion vectors (red), and accepted motion vectors (green) in two different cases: (**a**) forward motion and (**b**) change of the heading; (**c**) The number of the accepted motion vectors for two consecutive frames.

The matched interest points of two successive frames are determined using Euclidean distance between the descriptors of those points. Then, the candidate motion vectors are defined as the vectors starting form an interest point in one frame and ending at its corresponding matched point in the next frame. Figure 12 shows the matched features in two successive frames, candidate motion vectors (red), and acceptable motion vectors (green). As shown in Figure 12a,b, some of the matched points could be incorrect due to the existence of repeated similar points in the frames. Therefore, the candidate motion vectors should be filtered out to remove the inconsistent vectors. This filtering is based on discrepancy in length or orientation of the candidate vector. The RANSAC algorithm is used to find the compatible vectors using vector angle and length criteria. The accepted motion vectors are then averaged to make the average motion vector. The accuracy of the average motion vector depends on the number of the compatible vectors and variance of the angles and lengths of these vectors. Figure 12c shows the number of acceptable motion vectors from the first 20 motion vectors detected as the best matches in the successive frames.

Under the assumption of having context information of the hand-held device alignment (texting mode and landscape forward alignment), the vertical component of the average motion vector is a measure of the forward motion speed. The horizontal component of the average motion vector is a measure of the azimuth change between two successive frames. To calibrate the scale approximation between the motion vector and both the forward velocity and the azimuth changes, a reference track is navigated using the motion vectors only. The transformation parameters between the motion vector and forward speed and azimuth change are estimated, so that the navigation solution corresponds to the reference solution. Using the estimated scaling parameters, the forward motion velocity and the azimuth changes can be approximated between any two successive frames with the help of the average motion vector. The estimation of the velocity from camera is improved by a scale factor which changes adaptively based on the user mode such as walking, stairs, and running context information. Also the heading measurements from visual sensor are compensated for the cases which the orientation of the device is changing instead of the orientation of the user by using the device orientation context.

Relative measurements from the visual odometery algorithm tend to accumulate error over time, resulting in long-term drifts. To limit this drift, it is necessary to augment such a relative navigation system with global positioning system such as GPS.

### 6.2. Vision-Aided Pedestrian Navigation using Sensor Fusion Algorithm

The core of the vision-aided pedestrian navigation system consists of GPS position and velocity measurements as well as the position aid (velocity and heading change rate) provided from video image frames. These measurements are integrated using a KF filter [7] that is presented briefly in the following section. In this research, the dynamic system is defined based on a walking user while texting in an outdoor environment. In order to model the characteristics of the two-dimensional motion of a walking user, PDR algorithm is used. PDR is the determination of a new position from the knowledge of a previously known position, using the current distance and heading information. The coordinates (*E_t_*, *N_t_*) of a new position with respect to a previously known position (*E_t_*_−1_, *N_t_*_−1_) can be computed as follows:

(1) $${\left[\begin{array}{c} E\\ N \end{array}\right]}_{k}={\left[\begin{array}{c} E\\ N \end{array}\right]}_{k-1}+\alpha V_{k}\cdot \Delta t_{k}{\left[\begin{array}{c} \sin\psi \\ \cos\psi \end{array}\right]}_{k-1}$$

where *E*, *N* represent the absolute position in the *East* and *North* coordinate, both in meters, *V* (m/s) is the speed and *ψ* (radian) is the heading defined with the origin north and clockwise positive the origin. α is a scale factor for incorporating the user's context. In the scenario of this paper, it is equal to 1 while the user is walking. However, this factor can be adaptively estimated for the cases of running using the step frequency [22] or walking down/up the stairs. The absolute observations from GPS and measurements obtained from camera have been integrated using a KF. This filter uses the dynamic model to make a prediction of the state in the next time step. Then, it uses an observation model to compare the predicted and observed states. The dynamic model of a KF is: *x_k_* = *Φ_k_*_−1_*x_k_*_−1_ + *w_k_*_−1_; where, *x_k_* is the state vector, *Φ_k_*_−1_ represents the transition matrix that relates the state of a previous time to the current time, and *w_k_* is the process noise which is assumed to be drawn from a zero mean multivariate normal distribution with covariance *Q_k_* (*w_k_* ∼ *N* (0,*Q_k_*)) [36]. In this case, the dynamic equations for vision aided GPS is:

(2) $$E_{k+1}=E_{k}+V_{k}\cdot \sin\psi_{k}\cdot \Delta t_{k}+w_{1}$$

(3) $$N_{k+1}=N_{k}+V_{k}\cdot \cos\psi_{k}\cdot \Delta t_{k}+w_{2}$$

(4) $${\dot{\psi }}_{k+1}={\dot{\psi }}_{k}+{\ddot{\psi }}_{k}\Delta t_{k}+w_{3}$$

(5) $$\psi_{k+1}={\dot{\psi }}_{k}\cdot \Delta t_{k}+\psi_{k}+w_{4}$$

(6) $$V_{k+1}=V_{k}+w_{5}$$

(7) $${\ddot{\psi }}_{k+1}={\ddot{\psi }}_{k}+w_{6}$$

where *ψ̇* (radian/s) is the heading change rate and *ψ̈* (radian/*s*^2^) is the derivative of heading change rate. The variable *Δt* presents the time between two epochs. The state vector in this system is:

(8) $$x_{k}={\left[\begin{array}{llllll} E_{k} & N_{k} & {\dot{\psi }}_{k} & \psi_{k} & V_{k} & {\ddot{\psi }}_{k} \end{array}\right]}^{T}$$

To avoid linearization, the state transition matrix is defined here simplified as:

(9) $$\left[\begin{array}{cc} \begin{array}{c} 1\\ \begin{array}{c} 0\\ \begin{array}{c} 0\\ \begin{array}{c} 0\\ \begin{array}{c} 0\\ 0 \end{array} \end{array} \end{array} \end{array} \end{array} & \begin{array}{cc} \begin{array}{c} 0\\ \begin{array}{c} 1\\ \begin{array}{c} 0\\ \begin{array}{c} 0\\ \begin{array}{c} 0\\ 0 \end{array} \end{array} \end{array} \end{array} \end{array} & \begin{array}{cc} \begin{array}{c} 0\\ \begin{array}{c} 0\\ \begin{array}{c} 1\\ \begin{array}{c} \Delta t_{k}\\ \begin{array}{c} 0\\ 0 \end{array} \end{array} \end{array} \end{array} \end{array} & \begin{array}{cc} \begin{array}{c} 0\\ \begin{array}{c} 0\\ \begin{array}{c} 0\\ \begin{array}{c} 1\\ \begin{array}{c} 0\\ 0 \end{array} \end{array} \end{array} \end{array} \end{array} & \begin{array}{cc} \begin{array}{c} \sin\psi_{k-1}\Delta t_{k}\\ \begin{array}{c} \cos\psi_{k-1}\Delta t_{k}\\ \begin{array}{c} 0\\ \begin{array}{c} 0\\ \begin{array}{c} 1\\ 0 \end{array} \end{array} \end{array} \end{array} \end{array} & \begin{array}{c} 0\\ \begin{array}{c} 0\\ \begin{array}{c} \Delta t_{k}\\ \begin{array}{c} 0\\ \begin{array}{c} 0\\ 1 \end{array} \end{array} \end{array} \end{array} \end{array} \end{array} \end{array} \end{array} \end{array} \end{array}\right]$$

*Φ_k_* is approximated as a constant matrix at every time epoch *k*. General form of the observation model is: *z_k_* = *H_k_x_k_* + *v_k_*; where *z_k_*, is the observation vector, *H_k_* is the observation model which relates the state space into the observed space and *v_k_* is the observation noise which is assumed to be zero mean and Gaussian white noise with covariance *R_k_(w_k_* ∼ *N*(0,*R_k_)*).The number of measurements fed to the filter is varied on an epoch-to-epoch basis based on the availability of the sensors and its data rate. The non-availability situation of the visual aiding is based on the matching accuracy and was discussed in the computer vision section. The accuracy of the GPS sensor is also available on the android smartphones. The full-scale measurement vector (*z_k_*) is as follows:

(10) $$z_{k}=\left[\begin{array}{ccc} E_{\text{GPS}} & N_{\text{GPS}} & \begin{array}{cc} V_{\text{GPS}} & \begin{array}{cc} {\dot{\psi }}_{\text{cam}} & V_{\text{cam}} \end{array} \end{array} \end{array}\right]$$

The KF works in two phases: the prediction and the update. In the first phase, the filter propagates the states and state's accuracies using the dynamic matrix *Φ_k_*_−1_ and ${\hat{x}}_{k-1}^{+}$ (estimated in the previous epoch), based on this equation: ${\hat{x}}_{k}^{-}=\Phi_{k}{\hat{x}}_{k-1}^{+}$. Then the covariance matrix *P_k_* can be estimated using $P_{k-1}^{+}$. The usual equation to calculate $P_{\text{k}}^{-}$ is: $P_{k}^{-}=\Phi_{k}P_{k-1}^{+}\Phi_{k}^{T}+Q_{k-1}$. In the update phase the state is corrected by a blending of prediction solution with the update measurements based on ${\hat{x}}_{k}^{+}={\hat{x}}_{k}^{-}+{\bar{K}}_{k}(z_{k}-H{\hat{x}}_{k}^{-})$; where, *K̅* is the Kalman gain obtained by $\bar{K}=P_{k}^{-}H_{k}^{T}{\left(H_{k}\;P_{k}^{-}H_{k}^{T}+R_{k}\right)}^{-1}$. The update of the covariance takes place with this equation: $P_{k}^{+}=P_{k}^{-}-{\bar{K}}_{k}H_{k}P_{k}^{-}$.

### 6.3. Experiments and Results

The potential of the proposed method are evaluated through comprehensive experimental tests conducted on a wide variety of datasets using the back video camera of a Samsung Galaxy Note smartphone. A dataset with two combined user context was collected for testing the total context-aware and navigation solution. The user walked along the side-line of a tennis court in a close loop. During the loop, the user changed the placement twice before and after making turns which represents a very challenging situation for vision navigation. Using the classification algorithm, the system recognized the mode change and adapts the most suitable vision-based heading estimation automatically. Then, to accomplish vision-aided solution, images resolution was down-sampled to 320 × 240 pixels. The frame rate of 4 Hz was chosen to capture motions vectors for walking mode.

**Figure 13.**
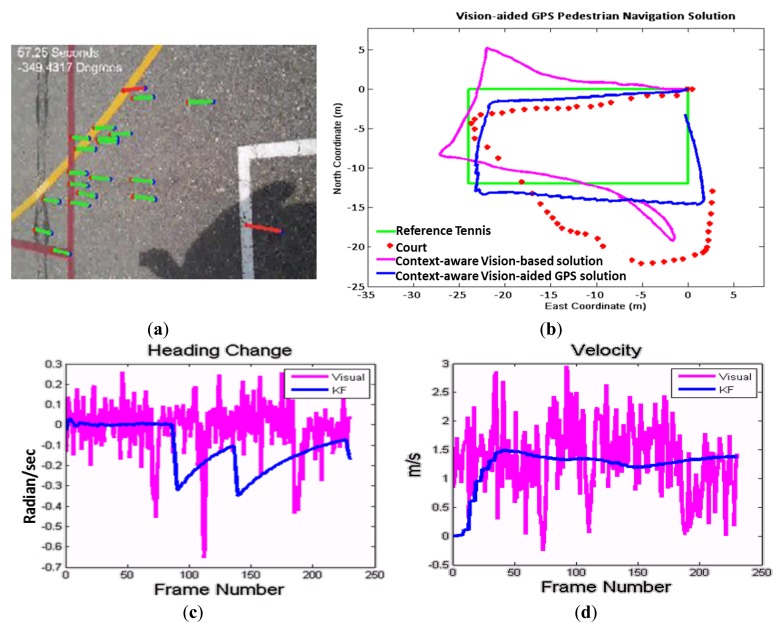
Vision aided GPS navigation: (**a**) An extracted video frame; (**b**) Vision-aided GPS in comparison with vision-based and GPS-only solution; (**c**) Heading angle changes estimated from visual sensor and KF; (**d**) velocity estimation using visual sensor and KF.

Figure 13a shows the extracted motion vectors, and Figure 13b shows the graphical comparison of the context-aware vision-aided GPS navigation using KF, vision-based navigation and GPS measurements. This figure shows that how vision-based measurements improve GPS navigation while both of them are not perfect enough in standalone mode. GPS solution in comparison with the vision sensor is not accurate enough and unable to discern turns. The reference trajectory is a tennis court located between two buildings and therefore, the smartphone's GPS navigation solution (red dots) has been degraded.

In Figure 13c, the KF and vision-based solutions are compared for the changes of heading estimation between two successive frames. In Figure 13d, the velocity estimation of the vision-based solution is compared with KF solution which is almost constant for a walking pedestrian.As previously shown for context-aware vision-aided navigation, the error of GPS navigation solution compared to the reference tennis court is reduced from 6.2 m in the case of using GPS only measurement to 2.7 m in the case of using vision-aided solution. By using context information, the vision-based algorithm can be aware of the appropriate user mode and the device orientation to improve and adapt it to the user's mode. To illustrate the usefulness of using context information for vision-based navigation, when the user was about to change his heading direction and turn 90° around the angles of tennis court, they were asked to change the device direction orientation from portrait to landscape (90° rotation). It means that the user heading changes about 90°, however the device heading changes about 90° ± 90° (“±” depends on the heading direction if it is clockwise or counterclockwise). Table 8 summarizes the navigation performance of the GPS navigation, a vision-aided navigation solution without considering context information and a context-aware vision-aided navigation which consider the change in device orientation from portrait to landscape (when the device heading changes about 90°). The experiment confirms previous findings where using the context information, the algorithm can distinguish between the orientation from user heading and device rotation. Therefore, context-aware velocity and orientation changes updates from visual sensor improve navigation solution in a smarter way.

As the smartphones are limited in terms of energy and computing power, another critical factor of the system performance is its computation time. For the context recognition procedure, a portion of computation including pre-processing is done on the mobile phone and the recognition process is performed on a central location or a server computer. Then, the results are sent back to the mobile phone for context-aware vision-aided solution. By evaluating time efficiency of different steps in context recognition, clearly the feature extraction is the most time consuming procedure. Table 9 describes the time budget of each step in context detection for activities recognition. Time efficiency in this table is obtained from a specific scenario of tennis court which is one minute sample data and in this case, optimum number of sensors and features are applied. However, it is useful for comparing the time efficiency achieved by this system. Sensor's signals are divided into two-second segments and features are extracted from 80 readings within segments. The preprocessing, noise reduction, calibration and segmentation of the signal are running on a Samsung Galaxy Note 1 which has a 1.4 GHz Dual Core Processor. The core signal processing and classification algorithms are written in Java programming language and running a CORE i7 CPU @ 2.7 GHz computer as a server. Also, the vision-based navigation solution is a real-time procedure running on the smartphone. To accomplish real-time vision-aided solution, images resolution was down-sampled to 320 × 240 pixels. The frame rate of 4 Hz was chosen to capture motions vectors for walking mode.

## 7. Conclusions and Future Work

The research investigates the design and development issues related to a context-aware personal navigation services for vision-aided navigation system. This paper contributes to the intelligent navigation computation domain using multi-level information fusion. A context-aware system is concerned with the acquisition of context (using sensors to recognize a context), the abstraction and understanding of context (modeling low-level context to infer about the user's situation), and adaptation of application behavior based on the recognized context. Since the context information may be acquired from multiple distributed and heterogeneous sources, defining an appropriate strategy to integrate various sources of information is necessary. To develop an approach for context recognition, two levels of fusion is used in this research: (a) Feature level fusion algorithms to combine data coming from different sensors by extracting useful features. (b) Decision-level fusion algorithms to detect high-level context information from multiple information sources and user constraints.

Feature-level context detection algorithm focuses on evaluation analysis of classifiers’ accuracy and providing reliable results for selecting the best set of sensors and features to optimize the performance of activity-logging applications on smartphones. Also extensive analysis was performed to investigate the effect of a separate estimation of user activity and device placement or considering both of them together. As an improvement to the previous works, accelerometer and gyroscope as well as other sensors such as GPS are integrated to recognize activity context more reliably. Moreover, no assumption has been considered for carrying mobile phone.

A high-level decision fusion is used to detect high-level context information from multiple information sources, such as user constraints and spatial-temporal dependency of recognized activities. This fusion algorithm contains three main steps: (i) Finding the rules for extracting activities, device status, and location; (ii) Collecting expert knowledge about the activity recognition and (iii) Implementing a rule-based system that includes a FIS for more reliable and readable results.

Finally, in this paper, a vision-aided pedestrian navigation algorithm is proposed to improve GPS solution. Based on the experimental results, the field test shows that texting mode (which is the proper mode for vision sensor) can be detected from accelerometer sensor with the accuracy of 82%. In this mode, the orientation of the device (*i.e.*, landscape or portrait mode) can be detected with an accuracy of 93%. Once context detection is performed, proper computer vision algorithm is applied accordingly to extract the motion vectors from successive frames. The motion vector is used to estimate user's motion. Then, in a sensor-level fusion algorithm, the GPS positions, velocity and vision-based velocity and the changes in heading angles are integrated. Pedestrian field tests were performed to verify the usefulness of the integrated algorithm. Context-aware vision-aided GPS solution outperform GPS only solution by 43%. The results are promising for combined vision-based GPS navigation and showed great potential for accurate, reliable and seamless navigation and positioning.

Although several important aspects of context-aware PNS using mobile devices and smartphones have been studied in this paper, there are still some open research problems worth further consideration. Overall classification performance can be improved by optimizing the methodologies based on the computing power. In this paper, data was only collected from four people, and a classification model was generated on top of this limited data set. To generate an adaptive model based on more training data from new users, it is interesting to have a larger population and build more useful and interesting applications. Also, considering other activities, such as bicycling, using transit, train or bus can be useful for PNS. Implementation of a robust context-aware PDR algorithm is another topic which is future research for the completion of on-foot navigation. This will include the appropriate changes in the KF states, prediction and update equations to integrate the PDR, wireless location estimation, vision and GPS solution for a ubiquitous indoor/outdoor navigation solution. Considering walking slow and fast, taking stairs and elevator for PDR and vision solution can be a useful adaptation for improving navigation solution.

## Figures and Tables

**Table 1.** Comparison of three different levels of fusion.

**Fusion Level**	**Advantages**	**Disadvantages**	**Application Examples**
Sensor level	Simple and real-time Problem independent Accuracy improvement	Sensor dependent Sensitivity to noise and sensor alignment	Location determination using Kalman filter, particle filter, *etc.*
Feature level	Less sensitivity to sensorial aspects	Necessity of finding optimum features and feature extraction	Activity recognition using Bayesian networks, support vector machine, *etc.*
Decision Level	Fusion of diverse type of information Robustness improvement	Problem specific solution Dependency on external knowledge	Context reasoning using Fuzzy reasoning, Bayesian decision theory, *etc.*

**Table 2.** The useful time and frequency domain features for context detection.

**Feature Space**		**Description**
Time-Domain	Mean	$\bar{y}=\frac{\sum_{i=1}^{N}y_{i}}{N}$ where *y_i_* are the samples, *i* = 1, …,*N*
	Standard Deviation	$SD=\sqrt{\frac{1}{N-1}\sum_{i=1}^{N}{\left(y_{j}-\bar{y}\right)}^{2}}$ where *y_i_* are the samples, *i* = 1, …,*N*
	Inter-axis Correlation	$R_{\vec{X},\vec{Y}}=\frac{n\left(\sum_{i=1}^{n}x_{i}y_{i}\right)-\left(\sum_{i=1}^{n}x_{i}\right)\left(\sum_{i=1}^{n}y_{i}\right)}{\sqrt{\left[n\sum_{i=1}^{n}x_{i^{2}}-{\left(\sum_{i=1}^{n}x_{i}\right)}^{2}][n\sum_{i=1}^{n}y_{i^{2}}-{\left(\sum_{i=1}^{n}y_{i}\right)}^{2}\right]}}$ where *x_i_* and *y_i_* are the samples from two axes, *i* = 1, …,*N*
	Zero-Crossing Ratio	$\text{ZCR}=\frac{1}{\text{N}-1}\sum_{\text{i}=1}^{\text{N}-1}I\left\{{\text{s}}_{\text{i}}{\text{s}}_{\text{i}-1}<0\right\}$ where s is a signal of length N and the indicator function 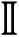 {A} is 1 if its argument A is true and 0 otherwise

Frequency-Domain	Frequency Range Power	$X\left(k\right)=\sum_{j=1}^{N}x\left(j\right)\omega_{N}^{\left(j-1\right)\left(k-1\right)}$ where *ω_N_* = *e*^(−2π^*^i^*^)/^*^N^* is the *N*th primitive root of unity(e.g., the frequency of walking is about 2 – 5 *Hz* [22], so, this frequency band separates activities such as walking and running)
	Spectral Energy	*S_xx_*(*ω*) = |*x̂*(*ω*)|^2^ (where *ω* is the angular frequency and *x̂* (*ω*) is Fourier Transform of the signal
	Spectral Entropy	*Entropy* = −Σ *P* (*x_i_*)log *P*(*x_i_*) where *x_i_* are the frequency components for a given band and *P*(*x_i_*) is the probability of *x_i_*

**Table 3.** Categorization of the classification methods.

**Classification Methods**	**Description**
**Naïve Bayes**	NB is a simple probabilistic classifier which uses Bayes’ theorem with naive independence assumptions. This assumption simplifies the estimation of *P*(*ActivityClass*|*features*) from the training data.

**Bayesian Network**	BN is a probabilistic graphical model that encodes probabilistic dependencies among the corresponding variables by using training dataset. BN learns relationships between activity classes and features to predict the class labels for a new sample.

**Artificial Neural Network**	ANNs are capable of “learning” by a number of known training patterns. In this research the used ANN has three layers; input layer, hidden layer and output layer. A simple back propagation algorithm (using RMSE) is used as the learning process.

**Table 4.** Selected feature using SVM and gain-ratio feature evaluator and their corresponding recognition accuracy (Classifier: BN).

**Recognition Scenario**	**Selected Feature Using SVM**	**Selected Feature Using Gain Ratio**
**Recognition of User Activity**	Mean	Mean
	Standard Deviation	Standard Deviation
	Spectral Energy	Frequency Range Power
	Frequency Range Power	Spectral Entropy
**Accuracy**	98.1%	97.3%

**Table 5.** Comparison of different classifiers in activity recognition of the DB of 120 min data using four essential features selected by SVM method.

**Classifier**	**Accuracy**	**Time**
Bayes Network	84.96	0.72
Naive Bayes Classifier	81.24	0.04
ANN(Multi-Layer Perceptron)	79.18	1.84

**Table 6.** Definition of fuzzy input variables [20].

**Linguistic Variables**	**Values**
Walking pattern correlation	*Proper (>0.6); Medium (>0.2 & <0.8); Poor (<0.4)*
Connectivity between activities	*High (<0.8); Medium (>0.4 and <0.7); low (>0.5)*
GPS DOP	*Good (1–4);Moderate (5–10); Fair (10–20);Poor (>20)*
GPS velocity	*Driving (>6 (m));Pedestrian (<8 (m))*
Temperature	*Cold (<17 °C); Normal (>17 and <27 °C);Hot (>27 °C)*

**Table 7.** Comparison of multi-layer fusion techniques’ accuracy.

**Method**	**Overall Accuracy (%)**
Feature-Level Fusion (BN)	84.96
Decision-Level Fusion (FIS)	43.0
Hybrid Method (integration of BN and FIS)	97.1

**Table 8.** Improvement in context-aware vision-aided GPS navigation in compare with GPS navigation.

**Navigation Solution**	**Root Mean Square Error (m)**	**Standard Deviation (m)**	**Minimum Error (m)**	**Maximum Error (m)**
GPS	6.2038	4.0522	0.9911	13.4886
vision-aided GPS	6.6798	5.7653	1.3135	16.1863
Context-aware vision-aided GPS	2.6798	0.7442	1.2857	4.0558

**Table 9.** Time efficiency of different steps in context recognition and vision-aided navigation.

**Procedure (for One Minute Sample Data)**	**Environment**	**Time (ms)**
Signal pre-processing	Mobile device	180
Feature extraction	Server	220
Classification	Server	50
Context reasoning	Server	260

**Procedure (for Processing One Image Frame)**	**Environment**	**Time (ms)**

Vision-based solution (Surf, Matching and finding motion vectors using context information)	Mobile device	200
Vision-aided GPS KF	Mobile device	5
